# Magnitude, trend, and causes of under-five mortality from Kilite-Awlaelo health demographic surveillance database, northern Ethiopia, 2009–2017

**DOI:** 10.1186/s12889-020-09554-z

**Published:** 2020-09-29

**Authors:** Hiluf Ebuy Abraha, Abate Bekele Belachew, Mohamedawel Mohammedniguss Ebrahim, Mengistu Hagazi Tequare, Mache Tsadik Adhana, Natnael Etsay Assefa

**Affiliations:** 1 College of Health Sciences, Ayder Comprehensive Specialized Hospital, Clinical Governance and Quality Improvement Unit, Mekelle University, Mek’ele, Tigray Ethiopia; 2 College of Health Sciences, School of Public Health, Mekelle University, Mek’ele, Tigray Ethiopia; 3 College of Health Sciences, School of Medicine, Mekelle University, Mek’ele, Tigray Ethiopia; 4 College of Health Science, Department of Midwifery, Adigrat University, Adigrat, Tigray Ethiopia

**Keywords:** Causes of death, KA-HDSS, Trend, Under-five mortality, U5MR, Verbal autopsy

## Abstract

**Background:**

Globally, neonatal and child mortality remains still high. Under-five mortality accounts for four-fifth of child and young adolescent deaths. In Ethiopia, though there has been a remarkable progress over the past years, under-five mortality is still high. Evidence from population-based longitudinal studies on under-five mortality is limited. Thus, this study aims to investigate the magnitude, trend, and causes of under-five mortality in the Kilite-Awlaelo Health Demographic Surveillance System, Northern Ethiopia.

**Methods:**

Kilite-Awlaelo health and demographic surveillance system was established in 2009 in the northern part of Ethiopia. Population-based longitudinal study design was carried out through extracting data for nine consecutive years (2009–2017). After smoothing the data revealed a visually decreasing trend. Linear, quadratic, exponential, and autoregressive time-series models were checked. Accordingly, the exponential trend model provided the best fit with the lowest standard error of estimate, lowest sum square error and highest adjusted R^2^ value. Cause-specific mortality was determined by cross tabulating cause of death with specific age death.

**Results:**

The overall under-five mortality rate was 35.62 per 1000 livebirths. The under-five mortality rate of rural and urban residents was 37.58 and 12.99 deaths per 1000 livebirths respectively. The exponential trend model showed the under-five mortality rate was declining exponentially. Bacterial sepsis 67(20.6%), prematurity 37(11.08%), intestinal infection disease 30(8.98%), acute lower respiratory infections 26(7.78%), and birth asphyxia 24(7.19%) were the major causes of under-five mortality.

**Conclusion:**

The overall under-five mortality rate for the surveillance period was comparatively lower. A statistically significant difference in under-five mortality rate was observed between urban and rural residents. A statistically significant declining trend in the under-five mortality rate was observed. Bacterial sepsis, prematurity, intestinal infection disease, acute lower respiratory infections, and birth asphyxia were the major causes of under-five mortality. We recommend the huge discrepancy in under-five mortality rate between urban and rural dwellers could be narrowed to some level by increasing healthcare access for rural residents.

## Background

Globally, child mortality remains still high. In 2017 alone an estimated 6.3 million children and young adolescents died, most causes being preventable childhood diseases. Under-five mortality accounted for 5.4 million (85.7%) of these deaths. Worldwide, in a 20 years period, between 1990 and 2010, significant improvements were noted in child survival with a 37% decrement in under-five mortality [1–3].

However, despite this substantial progress, in Sub-Saharan Africa the magnitude of child mortality reduction over the past 25 years was very low compared to other countries. Children continue to face extensive regional and income disparities in their likelihood of survival. Sub-Saharan Africa continues to be the region with the highest under-five mortality rate (U5MR) in the world. In 2017, the region’s average U5MR was 76 deaths per 1000 livebirths [2].

In Ethiopia, though there has been a remarkable progress over the past 20 years, evidence shows that under-five mortality is still high. The 2016 Ethiopia Demographic and Health Survey (EDHS) showed that the estimate of the U5MR was 67 deaths per 1000 livebirths. Similarly, the 2019 Ethiopia Mini Demographic and Health Survey (EMDHS) revealed that the U5MR was 55 deaths per 1000 livebirths [4, 5].

Different studies tried to investigate the top causes of under-five mortality. Accordingly, many of them found out that preterm birth complications, acute respiratory infections, intrapartum-related complications, congenital anomalies, and diarrhea were the predominant causes of under-five mortality [3, 6].

Undertaking this under-five mortality situation requires an extensive advancement in the coverage and quality of neonatal and child healthcare services in the country. Studies discovered that Antenatal Care (ANC) interventions [7] and health facility delivery [8] reduce neonatal and child mortalities in low and middle-income countries. It is, therefore, very important that pregnant women utilize ANC and health facility delivery. Additionally, postnatal care (PNC), which is the most neglected care, is also a very critical phase in the lives of newborn babies [9]. Nevertheless, ANC, health facility delivery and PNC follow-up are underutilized in Ethiopia. The EDHS 2016 showed that only 28% of mothers were delivered by a skilled provider and 26% were delivered in a health facility with only 17% PNC checkup in the first 2 days after birth [4].

The millennium and sustainable development goals are the two most important agendas, which played a big role in the under-five mortality reduction over the past two decades. The sustainable development goals (SDG), which were established to replace the millennium development goals in 2015, has 17 goals. The third goal is about ensuring healthy lives and promoting wellbeing for all at all ages and has goals within a goal, and the SDG3 aimed to reduce the global U5MR to at least as low as 25 per 1000 livebirths by the end of 2030 [10].

In line with the SDG, the Ministry of Health of Ethiopia has also applied multiple approaches and strategies to reduce child mortality and has prioritized five strategic transformation focus areas from 2016 to 2020. Improving the quality of care for maternal, neonatal and child health is one of the prioritized areas. The Health Extension Program (HEP), which is still under implementation, is another strategy and has improved newborn healthcare practices [11–13]. In Ethiopia, registration of vital events and national identity card proclamation was issued in August 2012 [14]. The law, which become compulsory in 2016, was not universal in its coverage as registration was restricted to Ethiopians until an amendment was made in 2017, which expanded the coverage of registration system to asylum seekers, refugees and non-Ethiopian citizens [15]. Reports showed that in the Ethiopian civil registration and vital statistics system, completeness of death registration is not available [16].

Health Demographic Surveillance system (HDSS) data are more generalizable and representative when it is combined through systematic comparison and triangulation with national or international data [17]. The main advantage of HDSS data are; it has very high statistical power, measures trend over time, and track real time data and therefore provide a clear picture of demographic and health progresses. So far in Ethiopia, a number of studies [18, 19] have been conducted regarding under-five mortality. Yet, evidence from population-based longitudinal studies on under-five mortality is limited. Additionally, there is no enough information regarding the trend of U5MR over time. Thus, the study was aimed to investigate the magnitude, trend, and causes of under-five mortality using population-based longitudinal data from the Kilite-Awlaelo Health Demographic Surveillance system (KA-HDSS) in Northern Ethiopia.

## Methods

### Study setting

This study was conducted at the KA-HDSS site, in Kilite-Awlaelo district, located in the eastern zone of Tigray, Northern Ethiopia. Kilite-Awlaelo is located around 802 km north of Addis Ababa, the capital city of Ethiopia. The KA-HDSS was established in 2009. The KA-HDSS, which became a member of the International Network of Demographic Evaluation of Populations and Their Health (INDEPTH) network in 2011, encompasses 10 administrative kebeles at baseline (A kebele is the smallest administrative component in the country), of which one is urban and nine are rural. After 2016, 2 additional urban kebeles were added to the HDSS. At the baseline of this open cohort a total of 14,455 households and 65,848 individuals, whose unique surveillance identification number was given, were collected through census and registered [20–22].

### Study design

Population-based longitudinal study design was carried out through extracting data for nine consecutive years (2009–2017).

### Source and study population

The source population was all children under-5 years of age (0–59 months) who lived in Kilite-Awlaelo district during the surveillance period. The study participants consisted of all under-five who were dwellers of the districts under surveillance and died from 2009 to 2017.

### Data collection tools and procedure

A standard structured interviewer-administered questionnaire was used to collect data every 6 months. Data collection was made continuously by trained data collectors who live in the study villages. A total of 36 data collectors, 3 supervisors, 1 data manager and 1 data clerk were involved in the HDSS. Data were collected from the KA-HDSS on a continuous open cohort-basis and 9 years (2009–2017) data were extracted retrospectively from the KA-HDSS database system. Causes of death in the population from registered deaths were identified and determined using Verbal Autopsy (VA). However, from 2016 to 2017, even though the data regarding the specific cause of death were collected by the VA interviewers, the VA information has yet not been interpreted by the physicians and, therefore, we didn’t determine the cause of death for the years 2016 and 2017. Verbal autopsy, which uses the International Classification of Diseases (ICD)-10 codes, is a standardized interview conducted with close relatives or others having detailed knowledge of the overall circumstances, signs, and symptoms leading to death. In case of under-five deaths, in order to decrease the possible recall bias about sign and symptoms reporting, information for the VA was collected from the mother or father of the baby or from close relatives with the best knowledge they could remember. Interviewed data were processed into likely medical causes of death by three physicians who were trained on applying ICD-10 and standardization procedures across other HDSS sites. First the two physicians assigned the probable cause of death independently using the information in the VA form, then, it was checked for agreement. Accordingly, if the probable cause of death assigned by the two physicians was not the same, the VA form was assessed by the third physician, as a tiebreaker, and decision was made based on two of the three physician’s agreement. However, in case three of the physicians made different diagnosis, the cause of death was labeled as undetermined. Thirty (8.98%) of the VA based cause of death in the analysis was undetermined.

### Data quality control

In the KA-HDSS, quality assurance measures were included in all features of the surveillance process. To maintain consistency, the questionnaire was first translated from English to Tigrigna (the native language of the study area) and was translated back to English. Training regarding the objective of the study, confidentiality of information, and techniques of conducting an interview was given to the data collectors and supervisors for five consecutive days. Incase of inconsistent or missing data detection during the data collection process, the questionnaire was returned to the data collectors for corrections. To maintain the quality of the data generated, continuous supervision was made by the research team and field supervisors and feedback was incorporated accordingly.

Furthermore, measures of quality assurance procedures were also applied to the data collection process that made use of the VA questionnaires for the deceased under-five children. VA was conducted using a standardize World Health Organization (WHO) questionnaire approved by INDEPTH for the under-five deaths occurring in the HDSS [22]. Through the data manager, finalized questionnaires with acceptable quality were given to data entry clerks to enter them into the main database, the Household Registration System (HRS.2). Upon completion of the data entry, data manager and data entry clerks checked to make sure that all the data were correctly registered in the main database. The data manager assured that all the data in the database were correct, consistent, and up-to-date [21].

### Data management and analysis

Data were entered into the HRS version 2.1 database on a regular basis. Then, data were exported into and analyzed using Stata version 15 statistical package. Frequencies and cross-tabulations were used to summarize descriptive statistics. The U5MR and its causes were the major statistical parameters computed. The U5MR was computed using the total number of deaths in under-five divided by the total number of livebirths and compute it in 1000 livebirths during the same period. A 95% confidence interval (CI) was also computed for the overall U5MRs. The levels of U5MR for each year starting from 2009 up to 2017 were calculated in the same manner and the line graph was designed for the trend at different years.

Since there was no obvious long-term upward or downward trend in the line graph plotted with the actual data, an exponential smoothing time-series model with 0.25 smoothing coefficient (weight) was employed (Eq. 1). A small value for the weight (0.25) was used since our goal was to smooth the influence of random effects and possible cyclical effects by eliminating unwanted cyclical and irregular variations in order to see the overall long- term tendency of the series. After the smoothing process, the data showed a slightly declining trend in U5MR. Since smoothing the data revealed a decreasing trend, four of the time-series models (Linear, Quadratic, Exponential and Autoregressive forecasting) were tested to find out which model best explained the trend. The rule of thumb was to select the model that best explained the trend-- i.e. the model with the lowest standard error of estimate and sum square error (SSE) and the highest adjusted R^2^ value was selected [23]. It appeared that the Exponential trend model provided the best fit. While, the linear model was the second most appropriate model the Autoregressive model clearly showed the poorest fit followed by the Quadratic model. The exponential trend model was therefore used to describe the trend (Table 1). Finally, cause-specific mortality, the mortality rate from a specified cause for a population, was determined by cross tabulating cause of death with specific age death.

**Table 1:** Comparison of time-series forecasting models, using Standard error of the estimate, SSE and Adjusted *R*^2^

Trend	Prediction Model	Standard error of the estimate	SSE	Adjusted R^**2**^	***P***-Value
Linear Trend	U5MR = 42.3–1.6(year code)	4.83	163.48	0.42	**0.036**
Quadratic Trend	U5MR = 39.69 + 0.652(year code) - 0.283 (squared year code)	4.81	138.74	0.42	0.080
Exponential Trend	U5MR = 42.76 (0.95^coded year^)	0.06	0.024	0.44	**0.030**
Autoregressive model (AR)	U5MR _(current year)_ = 31.29 + 0.10_(previous year)_	6.81	278.50	−0.16	0.850

### Exponential smoothing formula

1 $$ Current\ smoothed\ value=(W)\left( Current\ value\right)+\left(1-W\right)\left( Previous\ smoothed\ value\right) $$

### Ethical approval

The KA-HDSS site has received ethical clearance from the Ethiopian Science and Technology Agency, the Ethiopian Public Health Association (EPHA), the US Center for Disease Control and Prevention (CDC) and the Health Research Ethics Review Committee (HRERC) of Mekelle University. Head of a family or an eligible person among the family was interviewed to capture the occurrence of birth as well as death. Therefore, informed verbal consent was taken from the head of the family or eligible adult in the family. Data containing personal identifiers of subjects were not shared with third-party, to keep confidentiality.

## Results

### Socio-demographic characteristics

During the 9 years, from 2009 to 2017, a total of 11,593 livebirths were recorded, with 413 under-five deaths. Among the livebirths, 7306(63.02%) of them were health facility deliveries with 4185(36.10%) home deliveries. While 924(7.97%) of the livebirths were urban residents, 5924(51.10%) of them were males. Home was the common place of death for the under-five deaths accounting for 276(66.83%) of the total. The vast majority, 401(97.10%), of the under-five mortality occurred in the rural part of the study area. More than half, 229(55.45%) of the under-five deaths were males. Neonatal death accounted for nearly half, (46.73%), of the under-five deaths. Infant and child mortalities constituted 296(71.67%) and 117(28.33%) of the under-five mortality respectively (Table 2).

**Table 2:** Socio-demographic characteristics of the livebirths and deceased under-five, Kilite-Awlaelo HDSS site, Northern Ethiopia, 2009–2017 (*N* = 413)

Variable	Category	Frequency	Percent [95% CI]
Place of delivery of the livebirths	Health Facility	7306	63.02 [62.13–63.90]
	Home	4185	36.10 [62.13–63.90]
	Others	102	0.88 [0.72–1.07]
Residence of the livebirths	Urban	924	7.97 [7.48–8.48]
	Rural	10,669	92.03 [91.52–92.52]
Sex of the livebirths	Male	5924	51.10 [50.18–52.01]
	Female	5667	48.90 [47.97–49.80]
Place of death of the under-five	Home	276	66.83 [62.06–71.35]
	Health facility	121	29.30 [24.95–33.94]
	Others	16	3.87 [2.23–6.21]
Residence of the deceased under-five	Urban	12	2.90 [1.51–5.02]
	Rural	401	97.10 [94.98–98.49]
Sex of the deceased under-five	Male	229	55.45 [50.51–60.31]
	Female	184	44.55 [39.69–49.49]
Age at death of the under-five	Infantile period (< 12 months old)	296	71.67 [67.06–75.97]
	Childhood period (12–59 months old)	117	28.33 [24.03–32.94]

### Magnitude and trend of under-five mortality

The overall U5MR in the study period was 35.62 per 1000 livebirths with 95% CI [32.32, 39.16]. The U5MR was lowest in 2017 (24.83/1000 livebirths) and highest in 2011 (46.83/1000 livebirths). The observed U5MR has shown a dynamic distribution in the first 4 years, 2009–2012, but then a declining trend was observed over the last 3 years, 2014–2017. The U5MR between urban and rural residents was significantly different (*P*-value of 0.023), with 37.59 [95% CI 34.05, 41.37] per 1000 livebirths in rural dwellers and 12.99 [95% CI 6.73, 41.37] per 1000 livebirths in urban residents.

**Fig. 1:**
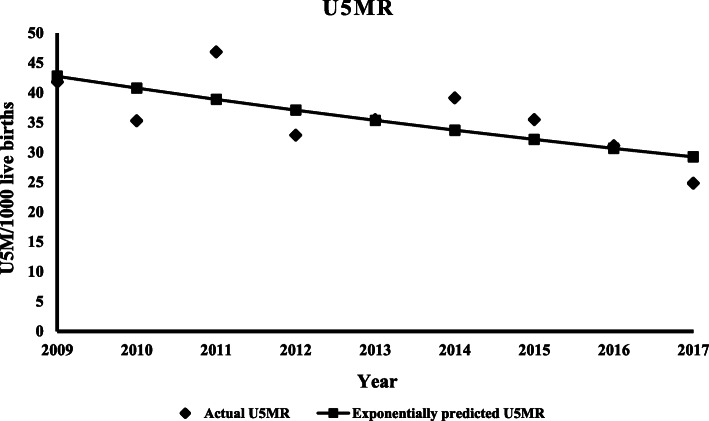
Under-five mortality rate trend, Kilite-Awlaelo HDSS, northern Ethiopia, 2009-2017

The exponential trend model provided the best fit with the lowest standard error of estimate (0.06), lowest SSE (0.024) and the highest adjusted R^2^ value (0.44). According to the Exponential trend time-series model, there was a statistically significant declining trend in U5MR. During the reference period U5MR was declining exponentially, U5MR = 42.76(0.95^coded year^), with an R^2^ value of 0.51, (*P*-value = 0.03), meaning about 51.1% of the variance in the U5MR can be explained by the trend (Fig. 1).

### Causes of under-five mortality

**Fig. 2:**
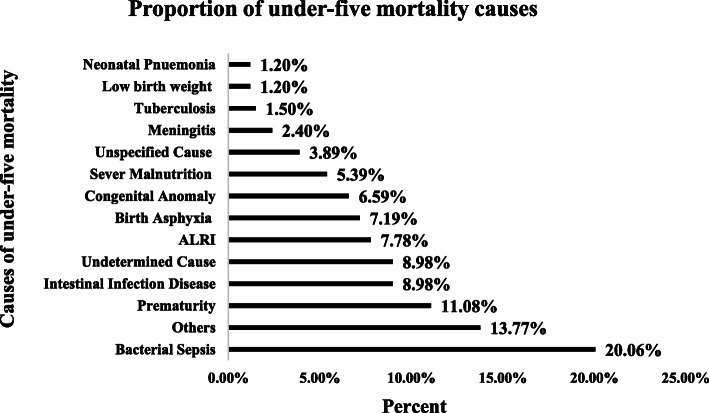
Proportion of causes of under-five mortality,  Kilite-Awlaelo HDSS, northern Ethiopia, 2009-2017

Causes of under-five mortality were analyzed for those whose cause of death was determined using the VA method. The VA was determined for the majority, 334(80.9%), of the death. Accordingly, based on the VA classification, the top five leading causes of death in under-five children were bacterial sepsis 67(20.6%), prematurity 37(11.08%), intestinal infection disease/diarrheal disease 30(8.98%), Acute Lower Respiratory Infections (ALRI) 26(7.78%), and birth asphyxia 24(7.19%). Others, like malaria, measles, epilepsy, asthma and other diseases related to the perinatal accounted for 46(13.77%) of the under-five deaths (Fig. 2).

## Discussion

This study aimed to assess the magnitude, trend, and causes of death among under-five children. Accordingly, the study revealed that the overall U5MR for the surveillance period, 2009–2017, was 35.62 [95% CI: 32.32, 39.16] per 1000 livebirths, with a statistically significant decline in the trend of U5MR. Among the causes of death, bacterial sepsis, prematurity and intestinal infection diseases were the top leading causes of death in under-five children.

The overall U5MR for the surveillance period was 35.62 [95% CI: 32.32, 39.16] per 1000 livebirths. This figure was in tune with a global U5MR estimated by United Nations International Children’s Emergency Fund (UNICEF) in 2017 [2]. However, our finding was lower than several studies done in Ethiopia [4, 5, 24, 25]. It is worth noticing that the overall magnitude in mortality and specific causes of under-five death were significantly influenced by neonates. Therefore, one possible explanation for the lower level of U5MR in our study could be partly explained due to the reason that health facility delivery was intensely higher in our study (63.02%), compared to multiple reports from Ethiopia [4, 5, 24–27]. This might have increased the care given to the neonates in their early period, thereby decreasing under-five mortality. Additionally, the present study was conducted on HDSS, and the population in such a setting could have better awareness about the health issues, since every household is visited every month for any event and the data are updated every 6 months.

A very huge discrepancy in U5MR between rural and urban residents was observed in our finding, with 37.58 per 1000 livebirths in rural dwellers and 12.99 per 1000 livebirths in urban residents. This significant difference was in agreement with reports from China, Vietnam, and Ethiopia [19, 28, 29]. This discrepancy in U5MR can be explained by a couple of reasons; first, there was unequal healthcare access between urban and rural residents, and health facility delivery was very high in urban dwellers (95.78%) than the rural residents (38.86%). Secondly, the fact that urban occupants accounted for only small proportion (2.90% of the deceased and 7.97% of the livebirths) of the subjects might have underestimated the mortality rate in the urban dwellers.

In this study there was a statistically significant declining trend in U5MR, which was in tune with an EDHS report [5]. The EDHS, which is conducted every 5 years, presented the U5MR trend starting 2005 to 2019, which showed a clear decline in the U5MR with 123 deaths per 1000 live births in the 2005 to 55 deaths per 1000 live births in the 2019.

Our finding established that neonatal death accounted for nearly half of the under-five deaths, which was in agreement with a 22 years trend analysis done in China, reports from WHO, and global systematic analysis [3, 30, 31]. In addition, similar to Demographic Health Survey (DHS) studies from Uganda and Ethiopia, our report also showed that more than two-third of the under-five deaths occurred during their infantile period [4, 5, 32].

In a couple of population-based epidemiological studies done in China, premature birth and birth asphyxia were among the top five leading causes of under-five mortality [28, 31]. Our study also identified premature birth and birth asphyxia to be the top causes of under-five mortality. Similar to our finding, previous researches from Nigeria and Ethiopia [18, 24, 33] have consistently found out that diarrheal disease was another leading cause of under-five mortality.

### Strength of the study

The KA-HDSS is the only surveillance system in the Tigray region, Northern Ethiopia. The fact that it is a longitudinal follow-up of the population and track real time data made it deliver a clear picture of demographic and health progress.

### Limitations of the study

Though more than 4/5th of the causes of death were collected from the VA information, the fact that we didn’t analyze the causes of death for the years 2016 and 2017 might have affected our study to a certain degree. Even though, efforts were made to decrease systematic error during extraction of the VA information, mothers or relatives of the deceased individual might have encountered recall bias when they were asked about the event. It is obvious that the VA can’t ascertain all causes of death, that’s why 8.98% of the VA based cause of death in our case was undetermined, which could be another limitation of our study.

## Conclusions

The overall under-five mortality rate for the surveillance period 2009–2017, was 35.62 per 1000 livebirths, which was lower than many studies done in Ethiopia. A statistically significant difference in U5MR was observed between urban and rural residents. Overall, a statistically significant declining trend in U5MR was observed. Bacterial sepsis, prematurity, and intestinal infection disease were the top causes of under-five mortality. Reports showed that health facility delivery played a huge role in decreasing neonatal mortality [8]. We, therefore, recommend the huge discrepancy in U5MR between urban and rural dwellers could be narrowed to some extent by increasing healthcare access for rural residents. This might increase health facility delivery of the rural occupants, which is only 38.86% this time.

## Data Availability

Our data source was the KA-HDSS database system, which we were allowed to access with the dataset after we obtained full permission from the office. The datasets generated and/or analyzed during the current study are not publicly available due to ethical and confidentiality reasons but are available from the corresponding author on reasonable request under the Ethics Committee’s approval.

## Abbreviations

ALRI: Acute lower respiratory infections
CI: Confidence interval
HDSS: Health and demographic surveillance system
HRS: Household Registration System
ICD: International classification of diseases
INDEPTH: International Network of Demographic Evaluation of Populations and Their Health
KA: Kilite-Awlaelo
U5MR: Under-five mortality rate
UNICEF: United Nations International Children’s Emergency Fund
VA: Verbal autopsy

## References

[1] WHO, UNICEF. Countdown 2015: building a future for women and children, the 2012 report. http://countdown2015mnch.org/documents/2012Report/2012-Complete.pdf. Accessed 5 Apr 2020.

[2] Hug L, Dharrow D, Zhong K, You D. Levels and trends in child mortality: report 2018: The World Bank; 2018. Available from: https://data.unicef.org/wp-content/uploads/2018/10/Child-Mortality-Report-2018.pdf.

[3] WHO (2017). Causes of child mortality: global health observatory data.

[4] Central Statistical Agency (CSA) [Ethiopia] and ICF (2016). Ethiopia demographic and health survey 2016: key indicators report.

[5] Ethiopian Public Health Institute (EPHI) [Ethiopia] and ICF (2019). Ethiopia mini demographic and health survey 2019: key indicators.

[6] WHO, Maternal and Child Epidemiology Estimation Group (2018). Child causes of death 2000–2017.

[7] Singh A, Pallikadavath S, Ram F, Alagarajan M (2013). Do antenatal care interventions improve neonatal survival in India?. Health Policy Plan.

[8] Tura G, Fantahun M, Worku A (2013). The effect of health facility delivery on neonatal mortality: systematic review and meta-analysis. BMC Pregnancy Childbirth.

[9] World Health Organization (2013). WHO recommendations on postnatal care of the mother and newborn.

[10] WHO (2018). Sustainable development goal three targets.

[11] FDRE, Ministry of Health. Health Sector Transformation Plan (HSTP): Federal Democratic Republic of Ethiopia; 2016. Available online: https://www.globalfinancingfacility.org/ethiopia-health-sector-transformation-plan-201516-201920.

[12] United States Agency for International Development (2012). Health care financing reform in Ethiopia: improving quality and equity.

[13] Karim AM, Admassu K, Schellenberg J, Alemu H, Getachew N, Ameha A (2013). Effect of Ethiopia’s health extension program on maternal and newborn health care practices in 101 rural districts: a dose-response study. PLoS One.

[14] Federal Democratic Government of Ethiopia (2012). Registration of vital events and national identity card proclamation no. 760/2012.

[15] Federal Democratic Government of Ethiopia (FDRE) (2017). A proclamation to provide for the amendment of vital events registration and national ID. No. 1049/2017.

[16] Center of Excellence for CRVS Systems. Snap shot of civil registration and vital statistics system of Ethiopia: International Development Research Centre; 2019. Available from: https://crvssystems.ca/sites/default/files/assets/files/CRVS_EthiopiaSnapshot_e.pdf.

[17] Bocquier P, Sankoh O, Byass P (2017). Are health and demographic surveillance system estimates sufficiently generalisable?. Glob Health Action.

[18] Deribew A, Tessema F, Girma B (2015). Determinants of under-five mortality in Gilgel Gibe Field.

[19] Woldeamanuel BT. Socioeconomic, demographic, and environmental determinants of under-5 mortality in Ethiopia: evidence from Ethiopian demographic and health survey, 2016. Child Dev Res. 2019;2019. 10.1155/2019/1073782.

[20] Vital events indicator and causes of death: from longitudinal datasets of HDSS and Addis Ababa mortality surveillance program in six Ethiopian public universities. 2013; Available: http://www.etpha.org/publications/other_publications.html?download=478:hdss-booklet. Accessed 17 Mar 2020.

[21] Mekelle University College of Health Science (MU-CHS) Profile. 2018; Available from: http://www.mu.edu.et/chs/index.php. Accessed 21 June 2020.

[22] International Network of Demographic Evaluation of Populations and Their Health (INDEPTH) Network: http://www.indepth-network.org/. Accessed 11 Mar 2020.

[23] Berenson M, Levine D, Szabat KA, Krehbiel TC. Basic business statistics: concepts and applications: Pearson Higher Education AU; 2012. p. 510–25.

[24] Assefa N, Oljira L, Baraki N, Demena M (2016). Health & Demographic Surveillance System Profile HDSS Profile: The Kersa Health and Demographic Surveillance System.

[25] UNICIEF (2015). Maternal and newborn health disparities, Ethiopia.

[26] Yaya Y, Eide KT, Norheim OF, Lindtjørn B (2014). Maternal and neonatal mortality in south-west Ethiopia: estimates and socio-economic inequality. PLoS One.

[27] Mekonnen Y, Tensou B, Telake DS, Degefie T, Bekele A (2013). Neonatal mortality in Ethiopia: trends and determinants. BMC Public.

[28] Zhang W, Chen D, Xu Y, Yang R, Zhao Z (2015). Mortality rate for children under 5 years of age in Zhejiang Province, China from 1997 to 2012. PLoS One.

[29] Lee HY, Do DV, Choi S, Trinh OT, To KG (2016). Trends and determinants of infant and under-five childhood mortality in Vietnam, 1986–2011. Glob Health Action.

[30] Liu L, Oza S, Hogan D (2015). Global, regional, and national causes of child mortality in 2000–13, with projections to inform post-2015 priorities: an updated systematic analysis. Lancet.

[31] Cao H, Wang J, Li Y, Li D, Guo J, Hu Y, Meng K, He D, Liu B, Liu Z, Qi H. Trend analysis of mortality rates and causes of death in children under 5 years old in Beijing, China from 1992 to 2015 and forecast of mortality into the future: an entire population-based epidemiological study. BMJ Open. 2017;7(9): 1-11. 10.1136/bmjopen-2017-015941 PMC5623503 28928178

[32] Uganda Bureau of Statistcs (UBOS) and ICF (2017). Uganda demographic and health survey 2016: key indicators report.

[33] Findley SE, Uwemedimo OT, Doctor HV, Green C, Adamu F, Afenyadu GY (2013). Early results of an integrated maternal, newborn, and child health program, Northern Nigeria, 2009 to 2011.

